# Urinary Incontinence and Overactive Bladder Symptoms in Women with Breast Cancer Being Treated with Oral Hormone Therapy

**DOI:** 10.1055/s-0040-1718440

**Published:** 2020-11-30

**Authors:** Rebeca Stahlschmidt, Amanda Canato Ferracini, Luana Moreira de Medeiros, Cinthia Madeira de Souza, Cassia Raquel Teatin Juliato, Priscila Gava Mazzola

**Affiliations:** 1 Universidade Estadual de Campinas, Campinas, SP, Brazil

**Keywords:** hormone replacement therapy, urinary incontinence, urinary bladder overactive, medication adherence, terapia de reposição hormonal, incontinência urinária, bexiga hiperativa, adesão ao tratamento

## Abstract

**Objective** The objective of the present study is to observe the frequency and severity of urinary symptoms in women with breast cancer (BC) being treated with oral hormone therapy, associating them to drug adherence.

**Methods** The participants were interviewed once from June to October 2016. The evaluation of urinary symptoms was performed by two questionnaires: International Consultation on Incontinence Questionnaire - Short Form (ICIQ-SF) and International Consultation on Incontinence Questionnaire Overactive Bladder Module (ICIQ-OAB). Adherence was evaluated by the Morisky-Green method. Statistical analysis was performed by the Mann-Whitney test, linear regression, and Spearman correlation.

**Results** Fifty-eight women were interviewed: 42 treated with tamoxifen and 16 with aromatase inhibitor. Twenty-seven women (46.5%) presented urinary incontinence symptoms and 15 (25.8%) presented stress urinary incontinence (SUI). Fourteen (24.1%) women had symptoms of overactive bladder (OAB). There was no statistical difference in symptoms between both treatments and duration of treatments. Higher scores in the ICIQ-SF questionnaire were associated with low/medium adherence and advanced age. Higher scores in the ICIQ-OAB questionnaire were associated with low/medium adherence.

**Conclusion** The present study showed a high prevalence of urinary symptoms, such as urinary incontinence and OAB, associated with low/medium adherence and older age in women with BC being treated with oral hormone therapy. Health professionals should be alert to these symptoms since it could influence life quality and adherence to treatment.

## Resumo

**Objetivo** O objetivo do presente estudo foi observar a frequência e a gravidade dos sintomas urinários em mulheres com câncer de mama em uso de terapia hormonal oral, associando estes com a adesão ao tratamento.

**Métodos** As pacientes foram entrevistadas uma única vez, entre junho e outubro de 2016. A avaliação dos sintomas urinários foi realizada por dois questionários: International Consultation on Incontinence Questionnaire - Short Form (ICIQ-SF, na sigla em inglês) e o Questionário Sobre Bexiga Hiperativa (ICIQ-OAB, na sigla em inglês). A adesão foi avaliada pelo método Morisky-Green. A análise estatística foi realizada pelo teste de Mann-Whitney, regressão linear e correlação de Spearman.

**Resultados** Foram entrevistadas 58 mulheres: 42 tratadas com tamoxifeno e 16 com inibidor de aromatase. Vinte e sete mulheres (46,5%) apresentaram sintomas de incontinência urinária (IU) e 15 (25,8%) apresentaram incontinência urinária por estresse (IUS). Quatorze (24,1%) das mulheres tinham sintomas de bexiga hiperativa. Não houve diferença estatística nos sintomas entre os tratamentos e a duração dos tratamentos. Os escores mais elevados no questionário ICIQ-SF estiveram associados à baixa/média adesão e à idade avançada. Os escores mais elevados no questionário da ICIQ-OAB foram associados à baixa/média adesão.

**Conclusão** O presente estudo mostrou alta prevalência de sintomas urinários, como IU e bexiga hiperativa, associadas à baixa/média adesão e à idade mais avançada em mulheres com câncer de mama em tratamento com hormonioterapia oral. Os profissionais de saúde devem estar atentos a esses sintomas, pois eles podem influenciar a qualidade de vida e a adesão ao tratamento.

## Introduction

Breast cancer (BC) is the most common and it has the highest mortality rate among women. 1 It can be staged according to tumor size, number of lymph nodes, and whether there is distant metastasis. 2 The tumor can also be positive for estrogen/progesterone receptors, which directs the treatment to an oral hormone therapy (HT) with tamoxifen or aromatase inhibitor (AI). Tamoxifen binds to the hormone receptor, reducing the effects of estrogen, thus, preventing the cell from multiplying. The other class of oral therapy, AI, prevents the peripheral conversion of androgens in estrogens. 3

Oral HT treatment has its advantages, since it consists of one pill a day, orally, being easy and practical to use. However, there are some side effects that may prevent full adherence to the treatment, like arthralgia or genitourinary symptoms. Some of the side effects are related to the decrease in estrogen levels and resemble menopause symptoms. The most common, in both treatments, are hot flushes, but each has its specificities according to the mechanism of action. 4 5 Among the genitourinary symptoms, there can be lower urinary tract symptoms (LUTSs), characterized by urgency, including overactive bladder (OAB) syndrome, with or without urinary incontinence (UI). There have been some studies that suggested a correlation between the overall decrease in estrogen and the occurrence of these symptoms. 6 7 8 9 The importance of identifying these symptoms and correlating them to the treatment can help the health team to make decisions about management, since the treatment will take at least 5 years, and requires the understanding and adherence of the women to the therapy. 10

The aim of the present study was to determine the frequency and severity of urinary symptoms in women with BC being treated with oral HT, associating them to drug adherence.

## Methods

### Settings

This was a cross-sectional study, conducted at the HT-exclusive dispensing pharmacy of a public university hospital, specialized in women's health at the University of Campinas (UNICAMP, in the Portuguese acronym), Campinas, SP, Brazil, located in the countryside of Brazil. The pharmacist or a technician dispenses monthly the hormone therapy (tamoxifen, anastrozole and megestrol) and an oral chemotherapy (capecitabine and cyclophosphamide) to the patient herself or to another responsible person. Thus, the women were invited to participate in the study when they attended at the dispensing pharmacy after having received their oral HT medication from a pharmacist or a technician. The participants were selected in the study from Monday to Friday, the dispensing pharmacy's time, between June and October 2016. All women signed the informed consent form before being included in the study.

The inclusion criteria were having agreed to participate in the study and having signed the consent form; being > 18 years old, with no age limitation restriction; having started to, or already being treated with tamoxifen or AI during the study period; being followed at the hospital and being available to participate in the interview in person. The exclusion criteria were not being legally capable, and women who have had history of treatment for OAB with medication or history of urinary incontinence (UI) symptoms. Demographic data was collected from medical records, and the questionnaires were applied. The present study was approved by the Research Ethics Committee (CAAE: 54977116.0.0000.5404).

Among the genitourinary symptoms evaluated were: (1) urinary incontinence defined as involuntary loss of urine, categorized in yes or no, presented in absolute numbers; (2) stress urinary incontinence (SUI), an urine leak that happens in situations with increased abdominal pressure, such as lifting weight, coughing and sneezing, 11 and (3) OAB syndrome, with urinary urgency, with or without UI, and no SUI, according to the International Continence Society (ICS) guideline. 6 11

### Questionnaires

The International Consultation on Incontinence Questionnaire (ICIQ) overactive bladder (OAB) and short form (SF) were applied, identified and evaluated the genitourinary symptoms in women with BC. Women who answered positively to question number 3, *“how often do you leak urine?”* were considered with UI. A higher score indicates more severe UI (range 0–21). Both questionnaires are recommended by the ICS and they were validated in Portuguese. 12 13 Women did not undergo diagnoses methods for UI and they did not take any medication to treat UI. Medication adherence was assessed by the self-reported Morisky-Green short version adherence questionnaire. It is a four-item questionnaire with high reliability and validity, which has been particularly useful in chronic conditions. Each item in the questionnaire was responded as 0, Yes and 1, No. The sum of four-item score indicates the level of medication adherence, classified in high, medium or low. 14

### Statistical Analysis

Statistical analysis was performed by the Mann-Whitney test, linear regression, with variables transformed to posts, adjusted for duration of treatment. The Spearman correlation coefficient was used for age and duration of treatment. Multiple analysis with stepwise were used to evaluate the correlation of urinary symptoms, type of HT, adherence, age, and duration of treatment (months after starting the hormone therapy). The SAS System for Windows (SAS Institute, Cary, NC, USA) was used to perform these tests. The significance value was 5%.

## Results

Fifty-eight women were interviewed: 42 (72%) women were treated with tamoxifen and 16 (28%) women with AI (15 with anastrozole and 1 with exemestane), all of which have agreed to participate in the study. The average age was 59 ± 12 years old in the tamoxifen group and 56 ± 11 years old in the AI group. Most women were Caucasian and had > 5 years of formal schooling. Eighty-nine percent ( *n* = 52) of patients did not smoke. The duration of treatment varied between groups and inside each group, with women both in the first and final months of treatment. High adherence was higher in the AI group. At the initial diagnosis, most diagnosed women had ductal invasive BC (36; 62%), classified as stage 0-II (39; 67%) and had hormone therapy (HT) as an adjuvant treatment (53; 91%) ( Table 1 ). Twenty-seven women (46.5%) presented UI symptoms, 15 women (25.8%) had SUI symptoms with involuntary loss “when I cough or sneeze” and 14 (24.1%) women had symptoms of OAB (characterized by answering “urge to urinate and have to run to the bathroom”). There were no associations with the presence of OAB and duration of treatment ( *p* = 0.6608). The ICIQ-SF scores in BC women using HT were between 11 and 17 from a maximum of 21, and the ICIQ-OAB scores were between 19 and 55 from a maximum of 56. When we consider a type of HT, 47% using tamoxifen and 44% using AI mentioned UI. According to the ICIQ-SF and ICIQ-OAB scores, there were no differences in the severity of SUI and OAB in the 2 groups ( *p* = 0.7534 and 0.9149) ( Table 2 ). The multiple analysis showed that women with higher scores in the ICIQ-SF questionnaire had low/medium adherence (odds ratio [OR] = 4.263; 95% confidence interval [CI]: 1.275–14.254, *p* = 0.0186) and were older (OR = 1.055; 95% CI: 1.000–1.112, *p* = 0.0491). No significant associations were found among type of HT (OR = 1.133, 95% CI: 0.347–3.706, *p* = 0.8360) nor duration of treatments (OR = 1.010; 95% CI: 0.982–1.040, *p* = 0.4846) with both ICIQ-SF and ICIQ-OAB scores, adjusted for age. Table 2 shows the symptoms percentages and questionnaires averages for each type of HT.

**Table 1:** Demographic data divided by hormone therapy

Characteristics	Tamoxifen ( *n* = 42)	Aromatase inhibitor ( *n* = 16)
Age (years old) (χ ± SD)	59 ± 12	56 ± 11
Ethnicity n(%)	
Caucasian	33 (78%)	15 (94%)
Noncaucasian	9 (22%)	1 (6%)
Schooling(years) n (%)	
≤ 8	22 (52%)	8 (50%)
≥ 9	20 (48%)	8 (50%)
Marital status n (%)	
With a partner	24 (57%)	4 (25%)
Without a partner	18 (43%)	12 (75%)
Smoking n (%)	
Yes/Ex-smoker	4 (10%)	2 (12%)
No	38 (90%)	14 (88%)
Physical activity (≥ 2x weekly) n (%)	
Yes	16 (38%)	8 (50%)
No	26 (62%)	8 (50%)
Histologic type n (%)	
Ductal invasive	27 (64%)	9 (56%)
Others	15 (36%)	7 (44%)
Previous treatment n (%)*	
Radiotherapy	29 (69%)	12 (75%)
Chemotherapy	32 (76%)	11 61%)
Stage	
0-II	31 (74%)	8 (50%)
III-IV	11 (26%)	8 (50%)
Type of treatment	
Adjuvant	40 (95%)	13 (81%)
Neoadjuvant	2 (5%)	3 (19%)
Duration of treatment (months) (χ month ± SD)	25 ± 15	17 ± 25
High adherence	17 (40%)	9 (56%)
Comorbidities n(%)	26 (62%)	9 (56%)
Hypertension	15 (36%)	2 (12%)
Diabetes	6 (14%)	1 (6%)
Dyslipidemia	3 (7%)	2 (12%)
Hypothyroidism	4 (9%)	1 (6%)

Abbreviations: χ ± SD - average ± standard deviation; HT, hormone therapy.

*In previous treatment, were included cases of women who underwent another therapy before beginning the HT.

**Table 2:** Symptoms and questionnaires divided by type of hormone therapy

Symptoms n (%)	Tamoxifen ( *n* = 42)	Aromatase inhibitor ( *n* = 16)	*p-value*
ICIQ-SF (±SD)	4.6 ± 6.1	3.4 ± 5.2	0.7534
SUI [n (%)]	11 (26%)	4 (25%)	
UI [n (%)]	20 (47%)	7 (44%)	
ICIQ-OAB (±SD)	11.0 ± 14.9	6.7 ± 7.6	0.9149
OAB [n (%)]	10 (24%)	4 (25%)	

Abbreviations: ± SD, average ± standard deviation; ICIQ-OAB, International Consultation on Incontinence Questionnaire Overactive Bladder Module; ICIQ-SF, International Consultation on Incontinence Questionnaire - Short Form; OAB, overactive bladder; SUI, stress urinary incontinence; UI, urinary incontinence.

## Discussion

The present study found a high prevalence on the sample of urinary symptoms in BC women using both HT treatments (tamoxifen and AI) and showed that high scores in urinary questionnaires were associated to incomplete adherence to HT, suggesting an association between severity of urinary symptoms and less adherence to treatment. Also, women from different ethnicities were considered across the place of study (Brazil), as it is a country of great miscegenation.

The present study found many women with urinary incontinence symptoms, with more than half having SUI. There was also a considerable number of women with OAB symptoms, higher than in a study with same geographic characteristics and age (average age 52.5 years old) that found 23.6% of women with UI, 6.4% with SUI and 7.8% with urinary urgency. 7 However, these numbers vary a lot considering the age groups. 15 16 The present study also found high scores of SUI and OAB, indicating the severity of symptoms in this population. Estrogen is the one female hormone that influences the voiding function of the bladder. Animal and epidemiologic studies related estrogen deficiency and OAB symptoms, because OAB could be explained as a storage symptom disorder. 9 As the purpose of oral HT is to decrease estrogen levels and its effect on BC tumor cells, it induces hypoestrogenism and therefore worsens the urinary symptoms. 7 8

These urinary symptoms were associated with older age and incomplete adherence. The symptoms can be affecting the way women see their treatment and how compliant they are. It was not correlated to time of treatment, indicating that these symptoms can appear at any moment, and not only if the woman is being treated for a long time. A large Taiwanese population study compared women from 18 to 40 years old with BC being treated with HT with a control group without cancer, and observed that the women being treated had higher incidence of OAB symptoms in the 1 ^st^ 3 years of treatment than the control group, indicating the role of estrogen in voiding function. 9 The present study compared two oral HTs, and observed the symptoms, with no difference between them, and in all times of treatment, with no difference as well.

Limited information about UI symptoms comparing the most used oral HTs increases the importance of the present research. The present study also aimed to collect some information on this topic at a reference women's health hospital. In this context, the present study reinforces the importance of a correct evaluation of the therapy side effects and the adherence of the patient.

The limitations of the present study include a difference between number of interviewed women in each group. The number of women being treated with tamoxifen on this study is three times bigger than that being treated with AI. So it is more probable to interview women in treatment with tamoxifen than with AI. Also, the interviews could only be performed during the time the researcher was at the distribution pharmacy and with the woman being treated, and not with other people that retrieved the medication with the prescription. Some demographic data was retrieved from medical records, and not from the interviewer. Another limitation is that the study design, being a cross-sectional study, prevents proving causality between urinary symptoms and HT. Future prospective studies are necessary to better establish this association. Pain and other side effects of the studied drugs are not considered in the present study. As with most BC studies and treatment, further studies with a control group, stratified by women without HT with a large sample size are necessary. Other studies considering all side effects of these drugs must be performed.

## Conclusion

The present study showed a high prevalence of urinary symptoms, including SUI and OAB, in women with BC being treated with HT. The severity of urinary symptoms was associated with incomplete adherence and age in women without difference between the two HTs. Health professionals should be alert to these symptoms, since they could be an influence in quality of life and compliance to this treatment.
